# Mitochondria and metabolic transitions in cardiomyocytes: lessons from development for stem cell-derived cardiomyocytes

**DOI:** 10.1186/s13287-021-02252-6

**Published:** 2021-03-12

**Authors:** Jessica C. Garbern, Richard T. Lee

**Affiliations:** 1 Department of Stem Cell and Regenerative Biology and the Harvard Stem Cell Institute, Harvard University, 7 Divinity Ave, Cambridge, MA 02138 USA; 2 Department of Cardiology, Boston Children’s Hospital, 300 Longwood Ave, Boston, MA 02115 USA; 3 Division of Cardiovascular Medicine, Department of Medicine, Brigham and Women’s Hospital and Harvard Medical School, 75 Francis St, Boston, MA 02115 USA

**Keywords:** Stem cells, Cardiomyocytes, Mitochondria, Maturation, Metabolic regulation

## Abstract

Current methods to differentiate cardiomyocytes from human pluripotent stem cells (PSCs) inadequately recapitulate complete development and result in PSC-derived cardiomyocytes (PSC-CMs) with an immature or fetal-like phenotype. Embryonic and fetal development are highly dynamic periods during which the developing embryo or fetus is exposed to changing nutrient, oxygen, and hormone levels until birth. It is becoming increasingly apparent that these metabolic changes initiate developmental processes to mature cardiomyocytes. Mitochondria are central to these changes, responding to these metabolic changes and transitioning from small, fragmented mitochondria to large organelles capable of producing enough ATP to support the contractile function of the heart. These changes in mitochondria may not simply be a response to cardiomyocyte maturation; the metabolic signals that occur throughout development may actually be central to the maturation process in cardiomyocytes. Here, we review methods to enhance maturation of PSC-CMs and highlight evidence from development indicating the key roles that mitochondria play during cardiomyocyte maturation. We evaluate metabolic transitions that occur during development and how these affect molecular nutrient sensors, discuss how regulation of nutrient sensing pathways affect mitochondrial dynamics and function, and explore how changes in mitochondrial function can affect metabolite production, the cell cycle, and epigenetics to influence maturation of cardiomyocytes.

## Introduction

Human pluripotent stem cells (PSCs) are capable of efficiently differentiating into beating cardiomyocytes within less than 2 weeks [1]. However, these PSC-derived cardiomyocytes (PSC-CMs) are immature, with characteristics that more closely resemble fetal cardiomyocytes than mature, adult cardiomyocytes [2]. Numerous strategies have been proposed to enhance maturation of PSC-CMs including electrical stimulation, prolonged time in culture, or use of patterned substrates with some success [2]. New studies reveal that metabolic stimuli such as glucose removal, fatty acids, or hormones can improve PSC-CM maturation [3–7]. These interventions recapitulate aspects of metabolic transitions that occur during embryonic, fetal, and postnatal development [8, 9]. In particular, birth itself triggers innumerable molecular changes as the body transitions from receiving all oxygen and nutrients from the placenta to breathing and eating independently. It is this window of time that cardiomyocytes transition from a proliferative state to a quiescent state [10, 11].

Common molecular mechanisms triggered by metabolic shifts may contribute to both cardiomyocyte cell cycle exit and maturation. In particular, mitochondria are central to both sensing and regulating transitions in nutrient availability [12]. Mitochondria also undergo maturation during the perinatal window, transitioning from small, fragmented organelles to large networks with developed cristae capable of the high oxidative capacity needed to produce enough ATP to support the contractility required of the postnatal heart [12]. This maturation process of mitochondria is likely not merely a response to overall cardiomyocyte maturation; rather, mitochondria may be mediators of the molecular processes triggering maturation of cardiomyocytes. Thus, understanding how metabolic transitions during development might affect mitochondria and how mitochondria can affect cardiomyocyte maturation is important to develop strategies to improve and accelerate the maturation process. In this review, we discuss the role that normoxia, enteral feeding, and hormone changes during the perinatal window can affect mitochondria; next, we highlight features of mitochondria that can be regulated by nutrient sensors; finally, we review how changes in mitochondria can regulate cell cycle activity, metabolite formation, epigenetic behavior, and electrophysiological properties of cardiomyocytes.

## Features of immature and mature cardiomyocytes

The features of immature, PSC-CMs compared to mature, adult cardiomyocytes have been reviewed extensively [2, 13, 14]. In brief, immature PSC-CMs have less organized and smaller sarcomeres (~ 1.65 μm), lower maximum contractile force, slower upstroke velocity, higher resting potential (around − 60 mV), absent T-tubules, and continued reliance on glycolysis as the primary energy source [14]. In contrast, mature, adult cardiomyocytes have more organized and longer sarcomeres (~ 2.2 μm), exhibit a lower resting membrane potential (around − 90 mV), and rely on oxidative phosphorylation for ATP production [2]. Mitochondria of immature PSC-CMs are small and distributed throughout the cytoplasm including many located in the perinuclear space, while mature mitochondria are larger and primarily intermyofibrillar or subsarcolemmal [2]. Prolonging culture time of PSC-CMs can increase mitochondrial content yet mitochondrial function and cardiomyocyte phenotype remain immature [15]. This raises a question reminiscent of the chicken and the egg dilemma: which comes first—mitochondrial maturation or cardiomyocyte maturation? Although both processes are intertwined, we focus here on mitochondrial responses to metabolic stimuli and consider their potential role as drivers of cardiomyocyte maturation.

## Overview of molecular sensors and mitochondrial responses

Molecular sensors detect changes in oxygen, energy, or nutrient levels to effect downstream transcriptional, translational, post-translational, or epigenetic modifications to enable responses to the environmental stimuli [16–19]. As one of the first organs to form during embryonic development, the heart must be able to utilize a variety of metabolic substrates in order to sustain its energy requirements throughout development. Cardiomyocytes retain this flexibility to utilize different substrates for ATP production, and shifts in the metabolic state can exert downstream effects that alter phenotype during development and disease states.

**Fig. 1:**
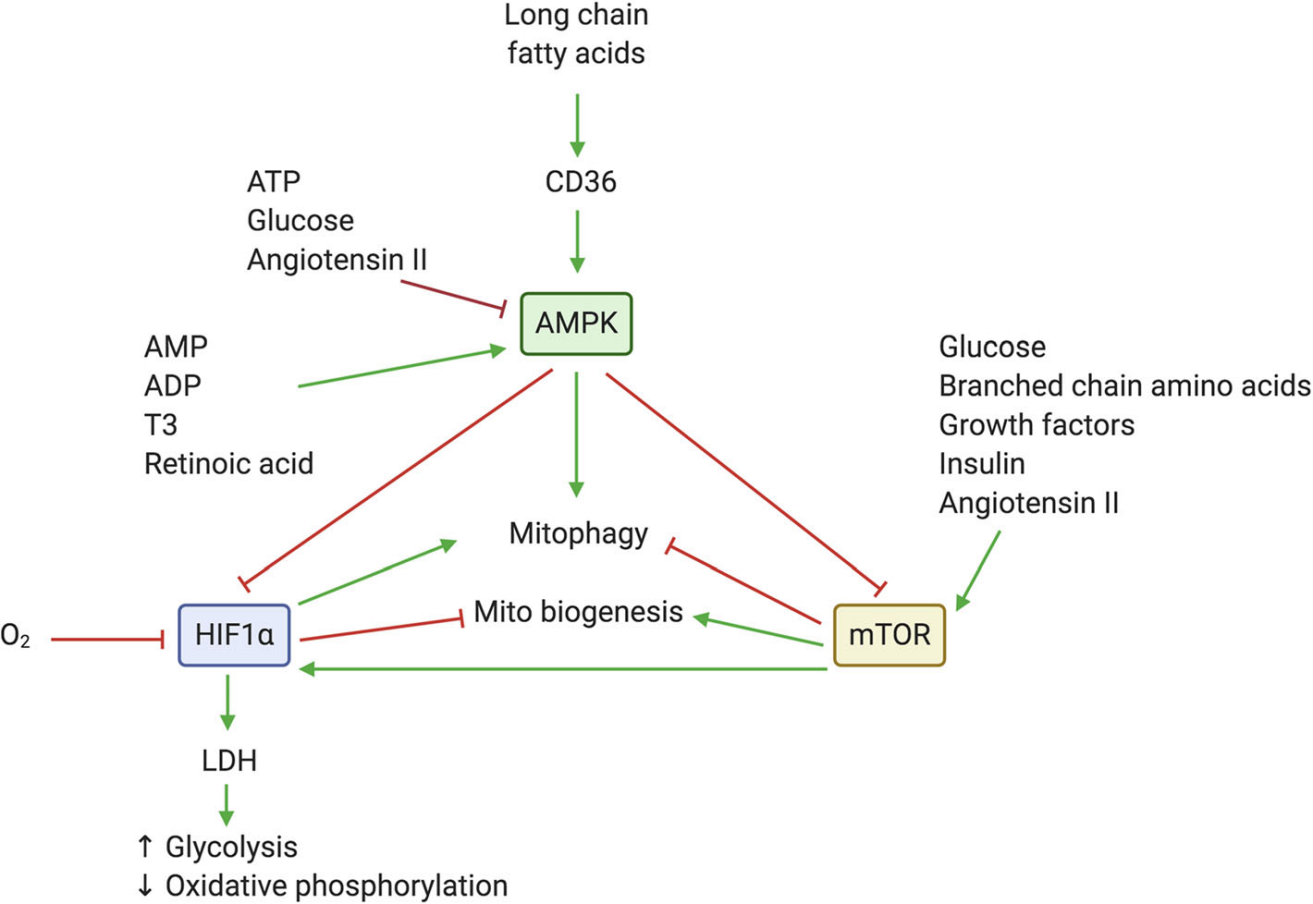
Metabolic sensors and mitochondria. Metabolic sensors detect changes in oxygen, nutrient, or hormone levels to induce or inhibit mitochondrial biogenesis or removal by mitophagy. Crosstalk between the AMP-activated kinase (AMPK), hypoxia-inducible factor (HIF), and mechanistic target of rapamycin (mTOR) signaling pathways can alter the balance of mitochondrial responses to nutrients or other metabolic signals

Major molecular metabolic/nutrient sensors in cardiomyocytes include hypoxia-inducible factors (HIFs; activated with low oxygen tension) [18], AMP-activated protein kinase (AMPK) (activated by low intracellular ATP levels in starvation conditions [20]), the mechanistic target of rapamycin (mTOR) (activated in nutrient-rich conditions [21]), and fatty acid receptors (e.g., CD36) [16]. Each of these systems has a close relationship with mitochondria, on whom they depend to transmit their signals to downstream processes (Fig. 1). In response to signals from these sensors, mitochondria can respond by changing the balance of mitochondrial fission and fusion, undergoing mitochondrial biogenesis, and/or initiating mitophagy to remove dysfunctional mitochondria. These mitochondrial morphologic changes have effects downstream by changing the oxidative capacity, mitochondrial membrane potential, and reactive oxygen species (ROS) levels to control cardiomyocyte phenotype [22]. We will discuss each of these further in the context of the different metabolic transitions below but briefly review their role in mitochondrial dynamics and function here.

Oxygen levels regulate activity of the HIF family of transcription factors, which are degraded under normoxic conditions and stabilized under hypoxic conditions [18] or can be activated by mTOR signaling [23]. HIFs consist of an oxygen-sensitive α unit (HIF-1α or HIF-2α) which dimerizes with HIF-1β to enact transcriptional changes aimed to promote cell survival in hypoxic conditions [24]. HIF-1α/2α suppresses oxidative phosphorylation by downregulating enzymes involved in the citric acid cycle to reduce acetyl-CoA formation and suppress electron transport chain activity in mitochondria, instead upregulating lactate dehydrogenase A (LDHA) to support glucose and lactate-mediated ATP production via glycolysis [24]. Interactions between HIFs and mitochondria have been reviewed previously—briefly, HIFs can downregulate mitochondrial biogenesis via crosstalk with AMPK signaling, differentially alter mitochondrial fission and fusion with acute or chronic hypoxia, alter expression of microRNAs that regulate mitochondrial function, and can upregulate antioxidant defense systems to minimize damage from ROS [25].

Nutrient sensors respond to alterations in glucose, amino acid, or fatty acid levels. AMPK is activated by increased levels of AMP and ADP, and as an energy sensor, it detects low glucose levels and initiates catabolic pathways to maintain energy homeostasis [20]. AMPK stimulates mitochondrial biogenesis and activates mitochondrial turnover via mitophagy by activating Unc-51-like kinase 1 (ULK1) [20]. The mTOR kinase acts through mTOR complex 1 (mTORC1) to increase protein synthesis and fatty acid metabolism and decrease autophagy or mTOR complex 2 (mTORC2) to promote cell survival [26]. mTOR signaling can be activated by glucose as well as amino acids and growth factors such as insulin and is negatively regulated by AMPK [16]. The fatty acid transporter, CD36, senses long-chain fatty acids and can activate AMPK [27]. In addition, it can modulate intracellular calcium levels or associate with the outer mitochondria membrane to regulate oxidative phosphorylation [27]. It is evident that molecular crosstalk between these metabolic sensors finely tunes the phenotypic response of cardiomyocytes in response to different substrates.

## Major metabolic transitions from fetal to postnatal life

Several excellent reviews have highlighted the many transitions that mammals undergo from embryonic formation to birth as they relate to cardiomyocyte maturation [8, 9, 13, 28]. Here, we focus on how these transitions specifically affect molecular nutrient sensors and how these sensors regulate mitochondria (Fig. 1). We use examples from both stem cells and development to underscore how mitochondria are much more than simply energy-producing organelles, acting to both listen to metabolic signals and coordinate cellular changes in response.

### Spontaneous breathing

Fetal development occurs in a state of relative hypoxia compared to the postnatal state, with fetal arterial pO_2_ of ~ 20–30 mmHg (versus adult alveolar arterioles pO_2_ of ~ 100 mmHg) [29]. At birth, there is a rapid increase in oxygen tension after the baby’s first breath which decreases expression of HIF-1α [18]. HIF-1α activity is mainly regulated by protein stability, with ubiquitination and subsequent degradation occurring rapidly in states of normoxia [30, 31]. In addition, lactate levels are relatively high in the fetus (~ 0.5–2 mM in umbilical venous blood at 17–21 weeks gestation [32]) due to high glycolytic activity of the placenta [33], which decreases rapidly after birth (~ 0.5–1 mM in umbilical venous blood at term delivery [32]) after achieving independence from placental circulation, with maternal arterial lactate levels of 0.68 ± 0.07 mM at delivery [34]. Lactate can also stabilize HIF-1α in fibroblasts [35] and cancer cells independent of hypoxia [36]. HIF-1α is required during embryonic development [29, 37], and deletion of HIF-1α in mouse embryonic stem cells (ESCs) reduces cardiomyocyte differentiation efficiency, with reduced expression of sarcomere proteins [38].

However, during later stages of differentiation, inhibition of HIF-1α has beneficial effects, enhancing contractile, electrophysiologic, and metabolic maturation of PSC-CMs and also leading to downregulation of LDH [3, 39]. These differential effects during early and late cardiomyocyte differentiation reflect the changing role of HIFs at the time of birth and during the perinatal metabolic switch. Because in vitro culture is usually performed in atmospheric conditions, small molecules or metabolic stimuli have been used to inhibit HIF-1α in culture (rather than manipulating oxygen levels). Treatment of human PSC-CMs with chetomin [3], FM19G11 [39, 40], or glucose-free, fatty acid-containing media [3] reduces HIF-1α and LDH expression and improves maturation of PSC-CMs. Hypoxia promotes a perinuclear distribution of mitochondria to promote ROS-mediated nuclear regulation [24]. Chronic hypoxia promotes mitochondrial fusion while acute hypoxia promotes mitochondrial fission [24]; thus, shifts in mitochondrial dynamics initiated by HIF-1α downregulation can alter mitochondrial function.

### Enteral nutrition and the metabolic switch

Initiation of enteral nutrition in the perinatal transition initiates a metabolic switch from glycolysis to oxidative phosphorylation [41]. This metabolic switch is not merely a feature of mature cardiomyocytes, but a key driver that initiates cardiomyocyte differentiation and maturation [42, 43]. The high fatty acid composition found in breastmilk likely facilitates this metabolic switch [8]. In addition, Let-7 microRNA (miRNA), which is also abundant in breastmilk [44], can induce a metabolic switch from glycolysis to oxidative phosphorylation and enhance PSC-CM maturation [45]. Here, we describe how shifts in glucose and fatty acids affect maturation of cardiomyocytes.

#### Glucose

The fetus depends primarily on glucose for energy, relying on the maternal-fetal glucose gradient for nutrient transfer [46]. In the first 24 h after birth, glucose levels are relatively low, with plasma glucose levels as low as 30 mg/dL (1.7 mM), and then glucose increases to levels similar to adults (fasting levels ~ 100 mg/dL or 5.6 mM) within the first few days of life after initiation of enteral feeding [47].

Pregnant mice or postnatal pups injected with ^18^F-labeled FDG (glucose analog) demonstrated decreased uptake of ^18^F-FDG in hearts at 1 and 7 days after birth compared to E10.5, suggesting that reduced glucose uptake in the heart postnatally may facilitate cardiomyocyte maturation in vivo [48]. During pregnancy, maternal hyperglycemia can lead to fetal cardiomyopathy and increase the risk of congenital heart disease [49]. Mice born to hyperglycemic mothers have asymmetric cardiac hypertrophy but with decreased cardiomyocyte size and decreased expression of TNNT2, suggesting that hyperglycemia promotes excessive proliferation and delays cardiomyocyte maturation during development [48].

PSC-CMs also demonstrate a decrease in ^18^F-FDG uptake relative to ^125^I-BMIPP (fatty acid analog) uptake at ~ 2 weeks of cardiomyocyte differentiation that increases further to at least week 8 of differentiation, indicating that PSC-CMs undergo a metabolic switch from using glucose to fatty acids during differentiation [50]. Standard media used for culture of PSC-CMs in vitro often has supraphysiologic levels of glucose (DMEM 4.5 g/L = 25 mM, DMEM/F12 31.5 g/L = 17.5 mM, RPMI 2 g/L = 11.1 mM glucose versus physiologic levels of ~ 5.5 mM). High glucose levels impair cardiomyocyte differentiation in both human [48] and mouse [51] ESCs, while upregulating genes associated with the cell cycle [48]. Suppressing glucose levels after cardiomyocyte specification enhances differentiation and maturation of PSC-CMs [3, 6, 48] by reducing biosynthesis of nucleotides via the pentose phosphate pathway [48]. Both AMPK and mTOR systems are exquisitely sensitive to changes in glucose levels, leading to downstream regulation of mitochondrial dynamics, discussed further below.

#### Fatty acids

During gestation, fetuses receive nutrition that is low in fatty acids from the placenta. In contrast, breastmilk has a high fatty acid content, and the composition of the fatty acids changes over time to match the developmental needs of the newborn [52]. There is an increase in fatty acid serum content from ~ 20 μM as a fetus to 300 μM as a newborn and 400 μM as an adult [53].

Sub-physiologic concentrations of fatty acids can enhance PSC-CM maturation, and PSC-CMs only uptake about 1/3 of fatty acids provided when at near-physiologic concentrations in culture [4]. Human PSC-CMs treated with 115.5 μM fatty acids (palmitate-albumin (52.5 μM), oleic acid-albumin (40.5 μM), and linoleic acid-albumin (22.5 μM)) for 2 weeks had increased size and force generation, increased mitochondrial respiratory reserve capacity, and increased action potential upstroke velocity compared to PSC-CMs not treated with fatty acids [4]. Similarly, PSC-CMs treated in glucose-free medium containing linoleic acid (9.4 μg/ml or 33.5 μM), oleic acid (9.4 μg/ml or 33.3 μM), and albumin (1 mg/ml) solution for 1 week exhibited more cell elongation, increased mitochondrial content, and increased basal oxygen consumption rate compared to control medium [54]. Palmitate (100 μM) alone increased MLC2v expression and force of contraction, and its effects were further increased in low-glucose (1 mM) solutions [6]. Without glucose, however, PSC-CMs cultured in fatty acid-rich medium experience lipotoxicity likely due to intracellular accumulation of fatty acids; addition of galactose (10 mM) can prevent lipotoxicity while further improving oxidative capacity and cardiomyocyte maturation [55]. By promoting oxidative phosphorylation by mitochondria, fatty acid availability directly impacts mitochondrial function and downstream metabolite formation.

#### Branched chain amino acids

The shift to enteral nutrition also changes the composition of plasma amino acids. The branched chain amino acids (BCAAs), leucine, isoleucine, and valine, are obtained through the diet and can activate mTOR signaling to regulate insulin signaling [56]. Physiologic levels of BCAAs may be facilitate cardiomyocyte maturation through stimulation of mitochondrial biogenesis, but excessive levels of BCAA may be detrimental due to overactivation of mTOR signaling. Hyperglycemia induces cardiomyocyte death in diabetic rats, and this may be due to abnormalities in metabolism of branched chain amino acids [57]. Hyperglycemia downregulates enzymes involved in catabolism of branched chain amino acids via inhibition of Krüppel-like factor 15 (KLF15), leading to intracellular accumulation of BCAAs, mTOR overactivation, and cardiomyocyte hypertrophy [58]. Chronic administration of water supplemented with branched chain amino acids can stimulate mitochondrial biogenesis, increase sirtuin 1 expression, and decrease ROS levels in cardiomyocytes while prolonging lifespan in mice [59]. Similarly BCAA supplementation can protect against doxorubicin-induced mitochondrial dysfunction and ROS production in cardiomyocytes [60].

### Hormone systems

Maturation of the endocrine system occurs simultaneously with cardiac development, leading to increases in fetal serum concentrations of hormones such as thyroid hormone or corticosteroids throughout fetal development. In addition, withdrawal of maternal estrogen after birth and alterations in retinoid signaling with enteral feeding may also play roles in cardiomyocyte maturation.

#### Insulin

Insulin levels are relatively high during the fetal state but decrease in the newborn before stabilizing to adult levels [53]. Insulin receptor and insulin-like growth factor 1 (IGF1) receptor facilitate cardiomyocyte proliferation starting at embryonic day ~ E10.5 [61]. In PSCs, early insulin (10 mg/L) treatment at the start of differentiation can inhibit cardiomyocyte formation, directing cells instead toward a neuroectoderm phenotype [62]. However, insulin does not inhibit cardiomyocyte differentiation induced by Wnt signaling, due to counteractive effects of β-catenin on insulin signaling [63]. Insulin/IGF1 treatment after cardiomyocyte formation increases proliferation and yield [64, 65], and combined IGF1 and neuregulin-1 (NRG1) treatment of PSC-CMs improves metabolic and contractile maturation [66]. Insulin activates mTOR signaling in the presence of glucose [67] and can also inhibit cardiomyocyte apoptosis [68] and promote mitochondrial fusion and enhance function by upregulation of OPA1 [69].

#### Triiodothyronine

Human fetal serum levels of total and free tri-iodo-L-thyronine (T3) and thyroxine (T4) and thyroxine-binding globulin increase throughout gestation, with total and free T4 and thyroxine-binding globulin levels reaching that of adults by 36 weeks gestation [70]. T3 serum levels remain well below maternal levels throughout gestation [70], with late fetal total T3 levels ~ 1–1.5 nM (65–98 ng/dL) and maternal levels ~ 2.5–3.5 nM (160–230 ng/dL), with mean non-pregnant adult levels ~ 2.1 nM (137 ng/dL) [70]. Congenital hypothyroidism is associated with cardiac defects, with 18.5% of congenital hypothyroidism infants having congenital heart disease in one study [71].

T3 has direct effects on cardiomyocyte development. T3 accelerates terminal differentiation and maturation of ovine fetal cardiomyocytes in vitro [72], and infusion of T3 during late gestation in sheep fetuses enhances cardiomyocyte binucleation, reduces cardiomyocyte proliferation (with increased p21 and decreased cyclin D1 expression), increases expression of SERCA2a, and increases cardiomyocyte size [73] via signaling through thyroid hormone receptors (TR) located in the nucleus [74]. Deletion of TRα1 in mice leads to bradycardia and reduced contractility with decreased myosin heavy chain α (MHCα) and SERCA2 expression, while deletion of TRβ1 in mice leads to tachycardia and no change in contractility [75]. T3 activates AMP-activated protein kinase (AMPK) leading to increased protein synthesis, while AMPK attenuates T3-induced cardiomyocyte hypertrophy in neonatal rat ventricular cardiomyocytes [76]. T3 rapidly induces an increase in AMPK activators (LKB1 (liver kinase B1) and CaMKK2β (calcium/calmodulin-dependent protein kinase kinase 2) and AMPK suppressors (PP2C (protein phosphatase 2C)), suggesting that narrow regulation of AMPK activity is required for normal cardiomyocyte development [76]. T3 can also reduce ROS following ischemia/reperfusion injury in mice and reduce H_2_O_2_-induced mitochondrial dysfunction and oxidative stress by increasing expression of the transcription factor, Nrf2, which increases expression of antioxidants such as HO-1, and activation of the PI3K/AKT signaling pathway [77].

Treatment of PSC-CMs with supraphysiologic levels of T3 (20 ng/ml = 2000 ng/dL = 30.7 nM) for 1 week starting after 20 days of differentiation increased cardiomyocyte size, increased expression of the cell cycle inhibitor p21, enhanced contractility, and increased oxygen consumption rate [5]. In another study, use of even higher levels of T3 (100 nM) for 2 weeks did not increase T-tubule formation unless combined with dexamethasone [78], thus synergy with other metabolic systems is likely necessary to observe maximal effects. Similarly, combined treatment with glucose-free medium containing T3 (10 nM) and fatty acids (linoleic and oleic acids) for up to 9 days exhibit a higher upstroke velocity, increased isoproterenol sensitivity, and decreased proliferation [79].

#### Glucocorticoids

Glucocorticoids are steroid hormones that act via the glucocorticoid receptor. Serum cortisol levels are low during mid-gestation (4 ng/mL at 17.5–20 weeks) but increase at term (45 ng/mL by 40 weeks) and increase sharply at delivery (mean 114 ng/mL in term infants at delivery) [80] before declining to basal levels (mean ~ 65 ng/mL in healthy infants) [81]. Global glucocorticoid receptor deficiency also prevents maturation of the heart in rats, with GR^−/−^ rats having disorganized myofibrils, reduced diastolic function, and fetal edema characteristic of hydrops fetalis [82], while maternal glucocorticoid administration prior to preterm piglet delivery enhances cardiomyocyte maturation [83].

Several treatment strategies have demonstrated PSC-CM maturation with dexamethasone. Dexamethasone (1 μmol/L = 392 ng/mL, 24–72 h) promotes electrophysiologic maturation of hESC-CMs with accelerated calcium transient decay, enhanced function of the Na^+^-Ca^2+^ exchanger (NCX) and sarco-endoplasmic reticulum calcium-ATPase (SERCA), and enhanced contractility [84]. Similarly, treatment of PSC-CMs with a combination of dexamethasone (1 μM) and T3 (100 nM), with [39, 85] or without [78] insulin-like growth factor 1 (IGF-1; 100 ng/mL) for 1–2 weeks promotes cardiomyocyte maturation. While glucocorticoids are important in normal cardiac development, they can also have detrimental effects in other models [86]—for example, chronic, supraphysiologic doses of glucocorticoids can increase the risk of cardiovascular disease in adults [87]—thus, further work is necessary to understand the optimal duration of treatment to maximize PSC-CM maturation without inducing deleterious effects of glucocorticoids.

The mechanism by which dexamethasone acts may be through mitochondrial maturation. Activation of the glucocorticoid receptor upregulates expression of PPARγ (peroxisome proliferator-activated receptor γ) coactivator 1α (PGC-1α) [88], a transcriptional coactivator that induces mitochondrial biogenesis and maturation [89]. Furthermore, short-term (24 h) dexamethasone treatment promotes maturation and inhibits proliferation of mouse ESC-CMs via mitochondrial aggregation of LC3 and induction of Parkin-mediated mitophagy [90]. Parkin-mediated mitophagy selectively degrades dysfunctional or depolarized mitochondria via autophagic processes activating the ubiquitin-proteasome system [91]. Thus, the combination of enhanced mitochondrial biogenesis, mitochondrial maturation, and elimination of dysfunctional mitochondria likely promotes glucocorticoid-induced cardiomyocyte maturation.

#### Estrogen

Estrogen levels are relatively high during fetal development due to maternal hormones, with estrogen withdrawal occurring within the days after birth [92]. Signaling through estrogen-related receptors α and γ (ERRα/γ) enhances maturation of cardiomyocytes during development [93], and ERRγ is involved in regulation of the metabolic switch to oxidative metabolism in the heart [94]. Deletion of ERRα/γ in mice leads to cardiomyopathy with arrested maturation of mitochondria, while ESRRA and ESRRA deletion in human PSC-CMs downregulates expression of non-cardiac genes, including those consistent with a fibroblast phenotype such as TCF21, periostin, and collagen type III [93].

#### Retinoic acid

Retinoids are the family of compounds derived from vitamin A [95]. The main biologically active metabolite, all-trans retinoic acid (ATRA) (also known as tretinoin), is formed by oxidation of retinol and signals via retinoic acid receptors (RAR) located in the nucleus [95]. RAR forms a heterodimer with retinoid X receptor (RXR) that then binds to retinoic acid response elements (RAREs) to regulate gene expression [96]. Serum ATRA concentration is lower in human newborns (3.4 nmol/L) than mothers immediately post-partum (5.8 nmol/L) and in control (non-pregnant or post-partum) women (5.2 nmol/L) [97].

Isolated deletion of RARα [98], RARβ [99], or RARγ [100] does not affect cardiac development, although double knockout of RARα with either RARβ or RARγ led to a high incidence of structural heart disease [101, 102]. Deletion of RXRα leads to ventricular failure during embryonic development [103]. Interestingly, excessive levels of vitamin A during development is teratogenic and can also increase the risk of congenital heart disease [104, 105]. RAR or RXR agonists prevent cardiomyocyte apoptosis and cardiac fibrosis induced by hyperglycemia in rat cardiomyocytes [106] via activating liver kinase B1 (LKB1) signaling and inhibiting p70 ribosomal protein S6 kinase activity (p70S6K) [107]. LKB1 is a key player in metabolic regulation through activation of AMPK, which can in turn inhibit the mTOR signaling pathway, and p70S6K is immediately downstream of mTOR complex 1 (mTORC1) [108]. Thus, retinoid signaling is closely tied to metabolic transitions that occur after birth with a shift in nutrient type and availability.

Treatment of PSC-CMs with supraphysiologic levels of ATRA (1 μmol/L) during early differentiation (days 2–4, mesoderm stage) increased expression of cardiac markers TNNT2, NKX2.5, and MYH6 by day 10, while treatment with retinoic acid from days 15–20 of differentiation enhanced cardiomyocyte size, increased action potential duration, and increased mitochondrial content and maximal respiration capacity [109]. Duration and dose of retinoic acid appear to be important factors in directing cardiomyocytes toward their desired phenotype, as treatment with 500 nmol/L ATRA from days 3–12 of differentiation leads to specification of an atrial cardiomyocyte phenotype rather than ventricular cardiomyocyte maturation [110].

#### Angiotensin II

Angiotensin II is a peptide hormone that regulates vasomotor tone and blood pressure and can enhance contractility [111]. Circulating levels of angiotensin II increase throughout fetal development and peak shortly after birth [112, 113]. Autoantibodies to angiotensin type 1 receptor (AT1R) during fetal development have decreased cardiac function with unorganized myofibrils and increased expression of proteins involved in glycolysis [114]. Crosstalk between the renin-angiotensin system and AMPK [115] may link angiotensin II to metabolic transitions occurring during cardiomyocyte development. Angiotensin II acts via the AT1R to promote differentiation of mouse ESC-CMs via c-Jun NH2-terminal kinase (JNK) and activation of the mitogen-activated protein kinase (MAPK) pathway leading to upregulation of sarcomere proteins and cardiomyocyte hypertrophy [116, 117]. Angiotensin II upregulates glucose uptake and mTOR signaling while downregulating AMPK activation leading to cardiac hypertrophy, while use of an AMPK activator increases fatty acid uptake and prevents cardiomyocyte hypertrophy [118]. These findings suggest that angiotensin II regulates early cardiomyocyte development through upregulation of sarcomere proteins but may have detrimental effects later by interruption of fatty acid metabolism.

### Noncoding RNAs

Noncoding RNAs, in particular microRNAs (miRNAs) (~ 18–24 nucleotides) and long noncoding RNAs (lncRNAs) (> 200 nucleotides), can be affected by changes in nutrient availability [119–121]. MicroRNAs (miRNAs) (18–24 nucleotides) can also regulate mitochondrial function, cellular quiescence, and maturation through epigenetic mechanisms involving post-transcriptional regulation of mRNA [25, 122]. A review of all miRNAs is beyond the scope of this review, but we highlight miR-1 as an example. Mice deficient in miR-1 have both structural and electrophysiological cardiac defects [123]. During differentiation of mouse ESC-CMs, miR-1 expression is upregulated, and overexpression of miR-1 can promote formation of cardiac mesoderm during early differentiation by suppression of WNT and FGF pathways [124, 125]. However, overexpression of miR-1 in adult rats increases the risk of ventricular arrhythmias by downregulating Kir2.1 (encoded by KCNJ2) and Cx43 leading to reduced conduction and cardiomyocyte depolarization [126]. While miR-1 can enhance ATP production in mitochondria, it is upregulated during senescence [127]. MiR-1 expression is increased in Hutchinson-Gilford progeria, a disease of accelerated aging [128]. While low levels of ROS (30 μM H_2_O_2_ × 2 days) can decrease miR-1 expression via ERK phosphorylation, high levels of ROS (200 μM × 6 h) can increase miR-1 via JNK phosphorylation in neonatal rat ventricular cardiomyocytes [129]. Furthermore, miR-1 exacerbates oxidative stress by reducing expression of antioxidant proteins in cardiomyocytes [130]. In addition, nutrient signals likely affect miR-1 expression, as inhibition of mTOR signaling in skeletal muscle represses miR-1 expression [131]. Hyperglycemia increases expression of miR-1 via p38 MAPK, leading to mitochondrial dysfunction, downregulation of Cx43, and cardiomyocyte apoptosis [132, 133], and these effects can be ameliorated by upregulation of liver X receptor α (LXRα) [134]. These studies demonstrate that fine tuning of multiple miRNA levels through nutrient availability is important to promote cardiomyocyte development.

In addition, long noncoding RNAs (> 200 nucleotides) are involved in both the pathophysiology of cardiac diseases [135] and cardiac development. For example, the lncRNA, *Hand2os1*, regulates the transcription factor, *HAND2*, to control cardiac morphogenesis during early development [136]. LncRNA expression is deregulated during states of metabolic stress such as in diabetes and altered expression patterns can be associated with development of diabetic cardiomyopathy [137]. Conversely, nutrient deprivation can acutely change expression levels of lncRNAs in skeletal muscle [138]. There are dynamic expression levels of lncRNAs in the heart, changing during the first week of life, suggesting an important role for lncRNAs in postnatal cardiomyocyte maturation [139].

### O-GlcNAcylation

O-GlcNAcylation is a post-translational modification analogous to phosphorylation that is regulated by nutrient availability and cellular stress [140–142] O-linked N-acetylglucosamine (O-GlcNAc) is a product of the hexosamine biosynthetic pathway that attaches to serine and threonine protein residues by O-GlcNAc transferase (OGT) and can be removed by O-GlcNAcase (OGA) [140]. Dysregulation of O-GlcNAc glycosylation adversely affects mitochondrial function [143]. Loss of OGT during development leads to dilated cardiomyopathy and abnormal coronary artery formation in mice [144]. OGT is required for maturation of postnatal cardiomyocytes by maintaining homeostasis of the endoplasmic reticulum and mitochondria [145] and is required for autophagy in cardiomyocytes [146]. However, excessive O-GlcNAcylation inhibits cardiomyocyte differentiation in ESCs [147]. Acutely increasing O-GlcNAcylation can be cardioprotective during ischemia in neonatal cardiomyocytes [148]; however, chronic elevation of O-GlcNAcylation as seen in diabetes leads to cardiac dysfunction and increase risk of arrhythmias via activation of Ca^2+^/calmodulin-dependent protein kinase II (CaMKII) [149, 150]. Thus, time-dependent or stage-specific regulation of O-GlcNAcylation may have opposing effects in cardiomyocytes.

## Metabolic regulation of mitochondria

**Fig. 2:**
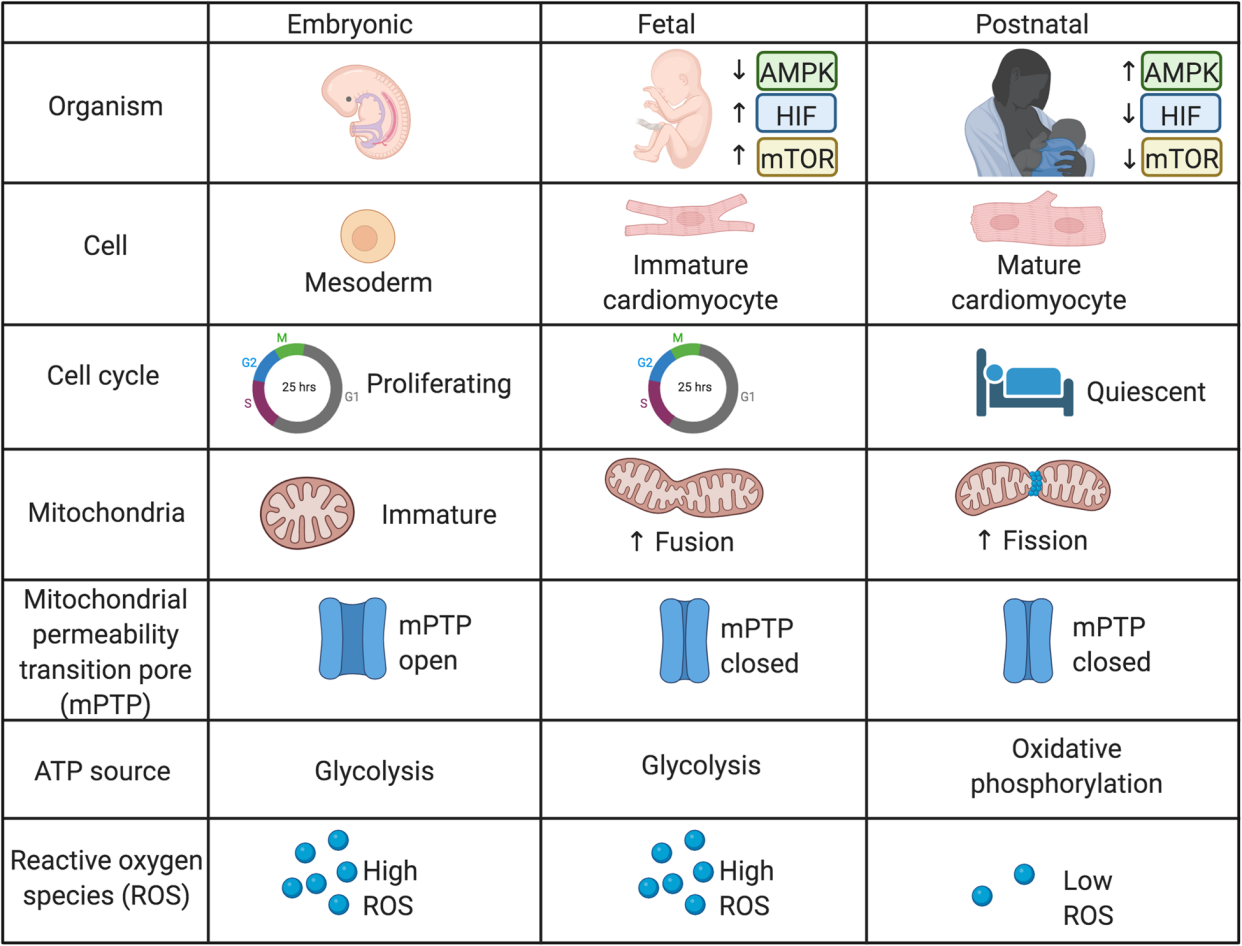
Metabolic transitions during development. Metabolic transitions from the embryonic to fetal to postnatal states regulate mitochondrial morphology and function, cell cycle state, cardiomyocyte maturation, and metabolite production

Given their central role in maintaining the metabolic phenotype, mitochondrial maturation may not simply be a byproduct of overall cardiomyocyte maturation but may actually drive cardiomyocyte maturation [151]. Metabolic shifts during fetal development and at birth target mitochondrial molecular pathways such as mitochondrial biogenesis, fission/fusion, mitophagy, and the mitochondrial permeability transition pore (Fig. 2).

### Mitochondrial biogenesis

Mitochondria in mature cardiomyocytes are large, occupying ~ 1/3 of the cardiomyocyte volume, and are organized between myofibrils (intermyofibrillar), beneath the sarcolemma (subsarcolemmal) or near the nucleus (perinuclear) [152]. In contrast, fetal mitochondria are smaller, have less well-defined cristae, and are poorly organized, distributed throughout the cytoplasm during early development, although they begin to relocate between myofibrils during later fetal development [152, 153]. Mitochondrial biogenesis that occurs during late fetal and early neonatal development is necessary to drive cardiomyocyte maturation [154].

Peroxisome proliferator-activated receptor gamma coactivators 1α (PGC-1α) and 1β (PGC-1β) are transcriptional coactivators that play key roles in mitochondrial biogenesis [154] and can be activated by AMPK [49]. PGC-1α^−/−^ or PGC-1β^−/−^ mice have mild cardiac dysfunction, but double deletion of PGC-1αβ^−/−^ leads to neonatal death with bradycardia, heart block, and cardiac dysfunction [154]. PSC-CMs upregulate PGC-1α during differentiation [155]. Activation of PGC-1α with ZLN005 improves maturation of PSC-CMs [89], while overexpression of PGC-1α in H9c2 cells stimulates mitochondrial biogenesis and autophagy to protect against lipopolysaccharide-induced apoptosis [156]. Contractile work requires increased ATP levels and this may also trigger enhanced mitochondrial biogenesis [157]. In addition, elevated glucose levels inhibit AMPK signaling which can adversely affect mitochondrial biogenesis and dynamics; activation of AMPK with berberine can reverse hyperglycemia-induced effects, leading to enhanced mitochondrial function by stimulating biogenesis and promoting mitophagy of dysfunctional mitochondria [158]. MiR-144 is decreased in hyperglycemic conditions, but overexpression of miR-144 can promote mitochondrial biogenesis and reduce apoptosis via AMPK phosphorylation and PGC1α deacetylation [159].

### Mitochondrial fission and fusion

**Fig. 3:**
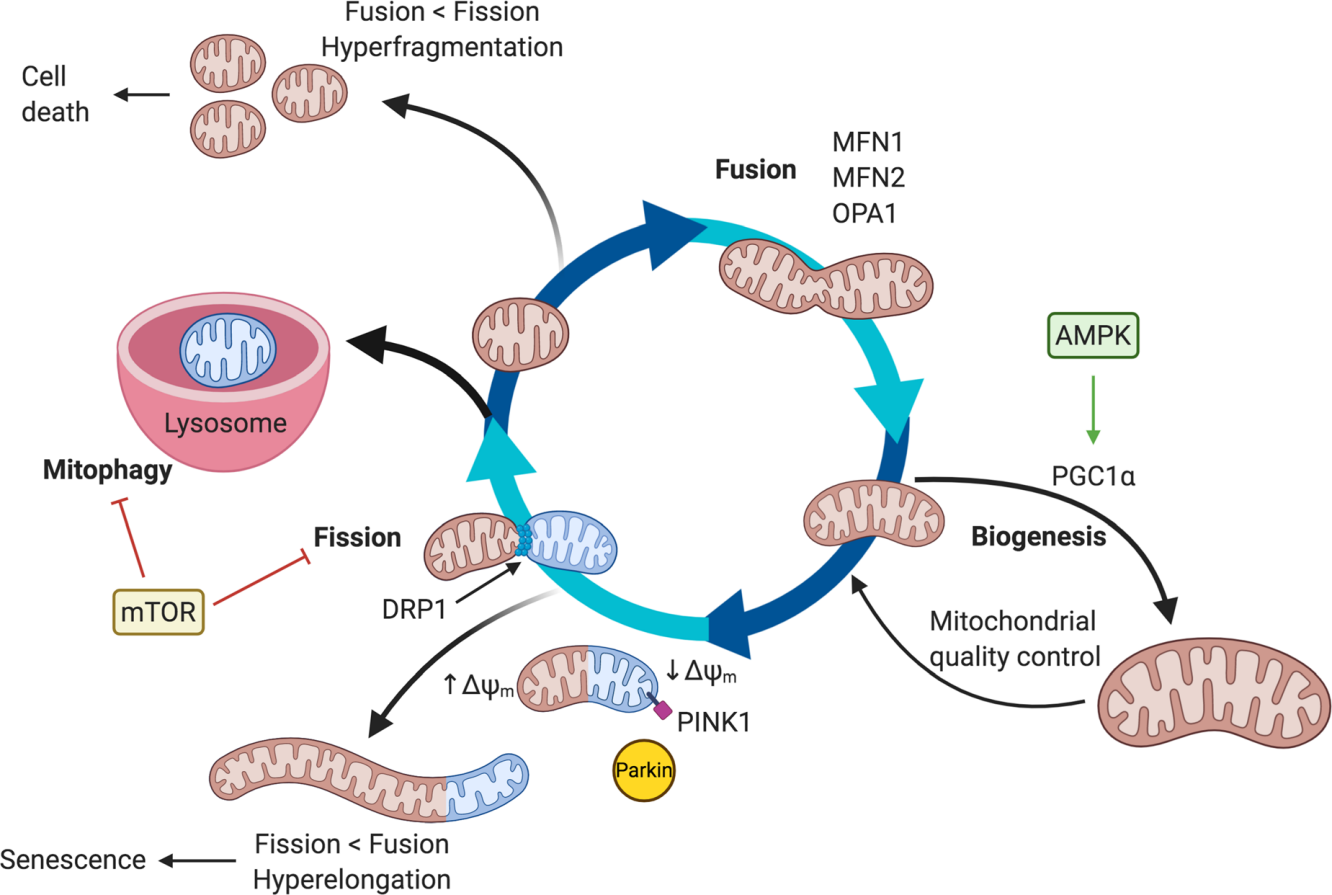
Mitochondrial dynamics. Mitochondria undergo dynamic processes of biogenesis, fusion, fission, and mitophagy. Balance of fission and fusion processes are needed to maintain normal mitochondrial function. An imbalance of fission/fusion leads mitochondrial dysfunction: excessive fission can lead to cell death and excessive fusion is often seen in senescence. Nutrient sensors AMPK and mTOR can regulate mitochondrial biogenesis and mitophagy

Mitochondria are dynamic organelles, undergoing processes of fusion and fission to maintain cardiac function (Fig. 3). Fission is regulated by PINK1/Parkin to signal the GTPase dynamin-related protein, DRP1 (encoded by *DNM1L*), to assemble into a constrictive ring to sever a mitochondrion into two halves [160]. Fusion occurs in two steps, first with fusion of the outer mitochondrial membrane (OMM) via mitofusins 1 and 2 (MFN-1/2), followed by fusion of the inner mitochondrial membrane (IMM) via interactions between optic atrophy 1 (OPA1) and cardiolipin [161]. In adult cardiomyocytes, mitochondrial dynamics are constrained by spatial limitations imposed by myofibrils [162], but changes in mitochondrial morphology are important in regulating developmental processes and in diseased states [163].

Isolated cardiomyocyte deletion of either *Mfn1* [164] or *Mfn2* [165] in mice promotes mitochondrial fragmentation that actually has a cardioprotective effect under stress conditions, but combined deletion of both Mfn1 and Mfn2 in cardiomyocytes is embryonic lethal by day E9.5 [166]. Similarly, cardiomyocyte deletion of *Dnm1l* in mice is lethal by 6 weeks of age [167]. Postnatal cardiomyocyte deletion of Mfn1/Mfn2 in mice leads to mitochondrial fragmentation and hypertrophic cardiomyopathy with left ventricular dilation leading to death within ~ 2–3 months [166], while Drp1 deletion leads to mitochondrial hyperelongation, cardiomyocyte necrosis, and a dilated cardiomyopathy phenotype with fulminant heart failure within 3–6 weeks leading to death [167]. Triple knockout of Drp1/Mfn1/Mfn2 remarkably delays the lethal effects of Drp1 deletion alone or Mfn1/Mfn1 deletion alone, exhibiting a concentric cardiac hypertrophy eventually leading to death between 3 and 6 months after tamoxifen treatment [168]. These findings demonstrate that an imbalance in fission and fusion processes causes more harm than when both processes are downregulated together.

OPA1 is a dynamin-like guanosine triphosphatase (GTPase) located in the inner mitochondrial membrane. Opa1 overexpression preserves mitochondrial function and prevents cardiomyocyte apoptosis after hypoxic injury through decreasing fission, increasing fusion, increasing mitophagy, and increasing mitochondrial biogenesis [169]. The full-length protein of OPA1 (long OPA1, or L-OPA1) facilitates mitochondrial fusion and is required during embryonic development [170]; however, OPA1 can be cleaved by two mitochondrial proteases, OMA1 or YME1L, to convert from L-OPA1 to a short form (S-OPA1), which reduces mitochondrial fusion and enhances fission [171].

During early differentiation, inhibition of fission and/or promotion of fusion may enhance differentiation efficiency, while promotion of fission may facilitate postnatal maturation. Mitochondrial elongation occurs during mouse ESC-CM differentiation, and downregulation of MFN2 or OPA1 prevents cardiomyocyte differentiation with reduced expression of Nkx2.5, Gata4, and Mef2c2 [172]. Furthermore, mitochondrial elongation prevents overactivation of calcineurin and Notch1 signaling to allow the transition from mesoderm to cardiomyocyte to occur normally, thus mitochondrial shape directly influences early cardiomyocyte development [172]. Promotion of fusion during PSC-CM differentiation increases the percentage of embryoid bodies that are beating and increases expression of cardiac genes [173, 174]. However, mitochondrial fission may be important in cardiomyocyte maturation during the neonatal period as mice deficient in Drp1 have disorganized myofibrils, reduced mitochondrial respiration, and abnormal cardiac function postnatally [175]. Wild-type mice have high expression of Drp1 in neonatal hearts at postnatal day 7 (P7) that decreases until mice are 4 weeks of age [175]; thus, a shift from a mitochondrial fusion to fission appears to accompany the metabolic switch that occurs postnatally and may be an important window for promoting cardiomyocyte maturation.

Mitochondrial fission and fusion processes are sensitive to intracellular and extracellular substrates; understanding how to fine tune the precise dynamics between the two processes is not well understood. Nutrients such as glucose or lipids can either activate or inhibit mitochondrial fission depending on context, and these processes are also regulated by post-translational modifications [176]. High glucose increases opening of the mitochondrial permeability transition pore (mPTP), ROS production, fission, and cell death [177–179]. However, removal of glucose of neonatal rat ventricular cardiomyocytes in culture can also increase Drp1 activation via S616 phosphorylation leading to mitochondrial fission and enhanced mitophagy [180] and reduced cell viability by 48 h [181]. While one interpretation of these in vitro results is that DRP1 activation in low-glucose conditions is detrimental to cardiomyocyte viability via increased autophagic processes, it may be that monolayer culture conditions in vitro affect the balance of mitochondrial dynamics, and Drp1 activity is inadequately balanced by fusion processes in vitro. Homozygous Drp1 deletion in adult mice leads to accumulation of elongated, dysfunctional mitochondria, reduced mitophagy, ventricular dysfunction, and death by ~ 3 months [180]. DRP1 activation may actually be a beneficial response under conditions of energy stress that is cardioprotective in vivo when fission and fusion processes are differently balanced compared to in vitro conditions. However, in streptozotocin-induced diabetic mice, inhibition of Drp1-mediated fission with melatonin improved mitochondrial function and reduced O_2_^−^ production in the heart via upregulation of SIRT1 and PGC1α [182]. Another theory is that DRP1 activation may be detrimental under conditions of chronic hyperglycemia while it may be beneficial in periods of acute fasting, thus culture of cells in high glucose media prior to the start of an experiment may affect the balance of mitochondrial fission and fusion processes that is different from physiologic conditions. Besides glucose, insulin can promote fusion [69], while a high-fat diet or elevated intracellular calcium levels increase fission [183, 184]. Nutrient shifts during the immediate postnatal period may have profound impacts on mitochondrial shape, which then can have downstream effects by altering mitochondrial biogenesis, mitophagy, metabolite production, and cell cycle regulation.

### Mitochondrial permeability transition pore

The mPTP is a conductance channel found in the inner mitochondrial membrane that permits equilibration of solutes less than 1.5 kDa in size [185]. During early heart development, mouse embryonic cardiomyocytes exhibit mitochondrial maturation between embryonic days E9.5 and E13.5, with closure of the mPTP, increased polarization of the mitochondrial membrane potential (ΔΨ_m_), decreased ROS levels, and development of more mitochondrial cristae [186]. Pharmacologic closure of the mPTP in E9.5 but not E11.5 or E13.5 myocytes using cyclosporine A (500 nM, 2 h) triggers mitochondrial maturation, resulting in mitochondrial elongation, increased ΔΨ_m_, and decreased ROS, as well as an increased number of myocyte Z bands suggestive of concomitant cardiomyocyte maturation [186]. Similarly, treatment of neonatal mouse cardiomyocytes with cyclosporine A to induce mPTP closure increased ΔΨ_m_, decreased ROS levels, and increased cardiomyocyte size, while treatment of neonatal mice from postnatal days 1–5 with cyclosporine A increased left ventricular ejection fraction [187], suggesting further maturation of cardiomyocytes occurs postnatally through this mechanism. Treatment of mouse or human ESC-CMs with cyclosporine A (2 μg/mL, days 4.5–10.5 of differentiation) or NIM811 (3.6 μg/mL), which also closes the mPTP, increased ΔΨ_m_, increased RNA expression of genes associated with cardiomyocyte differentiation and mitochondrial function, and increased the percent of TNNT2+ cardiomyocytes produced [188].

**Fig. 4:**
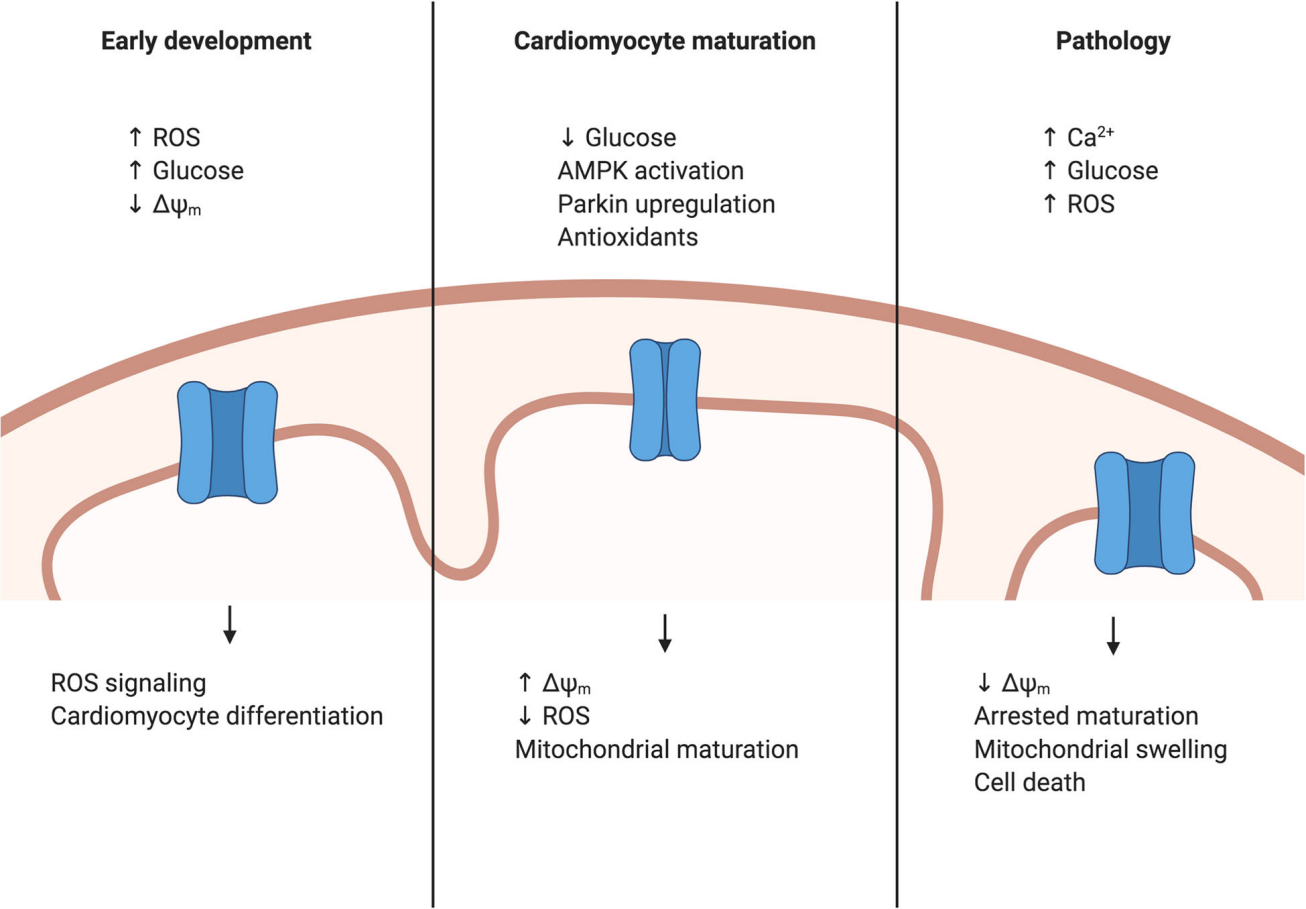
Mitochondrial permeability transition pore and cardiomyocyte development. During early development, the mPTP is open and increased ROS signaling facilitates cardiomyocyte differentiation. During later development, metabolic transitions trigger closure of the mPTP, which facilitates cardiomyocyte maturation. This is accompanied by an increase in the mitochondrial membrane potential and reduction in ROS, which also facilitate cardiomyocyte maturation. Abnormal opening of the mPTP can be triggered by elevated ROS, calcium, or glucose and can be seen in diseased states and may prevent cardiomyocyte maturation

The mPTP state can be affected by nutrient availability, calcium and ROS, and can affect insulin sensitivity. High calcium or ROS levels trigger mPTP opening, loss of ΔΨ_m_, mitochondrial swelling, and subsequent cell death [189]. Mitochondrial fusion proteins, MFN1 and MFN2, can promote mPTP opening [164, 165]. In a mouse model of ischemia reperfusion injury, inhibition of the nutrient sensor AMPK leads to mPTP opening during reperfusion, leading to increased ROS production and necrosis [190]. In addition, Parkin, a key regulator of mitochondrial mitophagy, can be upregulated by AMPK [191] and can also inhibit mPTP opening and prevent necrosis in cardiomyocytes by targeting cyclophilin D (CypD), a key component involved in opening of the mPTP [192]. Thus, targeted closure of the mPTP may be important for PSC-CM maturation (Fig. 4).

### Mitophagy

Autophagy of mitochondria, or mitophagy, is important to maintain mitochondrial quality control mechanisms through removal of dysfunctional mitochondria. The PINK1/Parkin system is responsible for initiation of this process: PINK1 accumulates in depolarized (dysfunctional) mitochondria, which recruits Parkin to the mitochondrial surface [193]. Parkin is an E3 ubiquitin ligase that ubiquitylates outer mitochondrial proteins to target it for proteasome degradation [194]. Deletion of Parkin between birth and weaning in mice prevents postnatal cardiomyocyte maturation, with impaired mitochondrial biogenesis and inhibition of fatty acid oxidation [195].

Mitophagy can be regulated by nutrient sensing systems such as Akt/mTOR signaling (upregulated in high nutrient states) or AMPK signaling (upregulated in fasting states) [196, 197]. Inhibition of mTOR signaling with simvastatin increases mitochondrial fission and mitophagy in HL-1 cardiomyocytes and is cardioprotective following myocardial infarction in wild-type mice [198]. Conversely, the AMPKα2 isoform phosphorylates PINK1 at Ser495 to remove dysfunctional mitochondria via enhanced mitophagy and protect against pressure overload in the heart [199]. Mitochondrial depolarization and subsequent mitophagy can also be caused by opening of the mPTP, which can be regulated by AMPK [167]. Upregulating fatty acid oxidation through deletion of acetyl coenzyme A carboxylase 2 (ACC2) promotes Parkin-mediated mitophagy and prevents myocardial dysfunction caused by a high-fat diet [200]. Thus the metabolic shift toward oxidative phosphorylation provides positive feedback to further hone mitochondrial quality control mechanisms through enhancing mitophagy. In contrast, inhibition of mitophagy via chronic activation of monoamine oxidase-A (MAO-A) leads to accumulation of cytosolic p53 and inhibition of Parkin, with subsequent reduction in mitophagy and retention of dysfunctional mitochondria [201]. MAO-A activation also leads to upregulation of cell cycle inhibitors, induction of the ROS-induced DNA damage response, and increased activity of senescence-associated β galactosidase (SA-β-gal), consistent with a senescent phenotype. Maintaining mitophagy may inhibit senescence and promote cardiomyocyte maturation.

## Molecular consequences of mitochondrial transformations

### Reactive oxygen species

Reactive oxygen species (ROS), including superoxide anion (O_2_^−^), hydrogen peroxide (H_2_O_2_), and hydroxy radical (•OH), are produced during oxidative phosphorylation and can have downstream signaling effects in cardiomyocytes via redox effects [202]. At physiologic levels, oxidation of selected proteins can maintain normal cellular function, but in excess, nonspecific oxidation of cellular contents can be toxic [203]. Oxidation of miRNAs such as miR-1 can lead to their inactivation and subsequent dysregulation of mRNA expression in the heart [204]. The role of ROS signaling during cardiomyocyte differentiation and maturation is context-dependent. During early differentiation of mouse ESC-CMs, transient increases in ROS with H_2_O_2_ or menadione improves cardiomyocyte differentiation with enhanced expression of sarcomere genes and improved contractility [205, 206]. Similarly, elevated glucose levels (25 mM) increases ROS formation in early PSC-CM differentiation compared to physiological levels (5 mM), and high glucose and high ROS promote cardiomyocyte differentiation, while low-glucose or early antioxidant treatment prevents cardiomyocyte differentiation [206, 207]. Upregulation of PGC1α during PSC-CM differentiation increases mitochondrial size and respiratory capacity while also increasing ROS production [208]. However, in mouse embryonic cardiomyocytes at embryonic day E9.5, antioxidant treatment enhances while oxidant treatment impairs cardiomyocyte differentiation [186]; this developmental stage also coincides with closure of the mPTP [186]. Furthermore, while knockdown of PGC1α impairs mitochondrial function, the concomitant reduction in ROS actually increases the action potential amplitude and duration closer to those seen in mature adult cardiomyocytes [208], possibly by preventing ROS-induced decreases in Ca^2+^ influx [209] or increases in K^+^ efflux [210]. Thus, the effects of ROS appear to be time-dependent: early ROS production may facilitate initiation of cardiomyocyte differentiation and sarcomere formation, while reduction of ROS levels after cardiomyocyte formation may be beneficial in maturing the electrical properties of cardiomyocytes.

### Cell cycle arrest

Mitochondria play integral roles in regulation of the cell cycle [211], while cell cycle state can control mitochondrial behavior [212]. Cardiomyocytes exit the cell cycle shortly after birth coinciding with a period of cardiomyocyte maturation [10]. The shift to oxidative phosphorylation increases ROS production by mitochondria after birth, and ROS scavenging can prolong the proliferative potential by postnatal cardiomyocytes [213]. A positive feedback loop exists between ROS levels, p38 MAPK and connexin 43 (Cx43), with increased ROS promoting expression of p38 MAPK and Cx43, and downregulation of p38 MAPK or mitochondrial Cx43 reducing ROS levels [213]. Increased ROS levels cause oxidative DNA damage that initiates a DNA damage response pathway that leads to cell cycle arrest in cardiomyocytes, while antioxidant treatment prolongs postnatal proliferation capacity [214]. Consumption of milk low in fatty acids also prolongs the postnatal proliferative capacity of cardiomyocytes, while deletion of pyruvate dehydrogenase kinase 4 (PDK4) to enhance glucose utilization and reduce fatty acid oxidation reduces DNA damage and cardiomyocyte size while also increasing proliferation [215]. These results suggest that the change to enteral consumption of breastmilk high in fatty acids both facilitates cardiomyocyte cell cycle arrest and maturation.

Additional regulators can affect both the cell cycle and the metabolic switch in cardiomyocytes. MEIS1, a transcription factor that stimulates cell cycle activity in cardiomyocytes [216], also supports a less mature metabolic phenotype in cardiomyocytes by promoting glycolysis, while MEIS1 siRNA in fetal sheep cardiomyocytes triggers a metabolic switch from glycolysis to oxidative phosphorylation [217]. Deletion of endonuclease G, a mitochondrial enzyme that promotes apoptosis during oxidative stress, increases ROS levels, reduces proliferative capacity of cardiomyocytes in mice, and increases cardiomyocyte size, with cell cycle arrest in the G1 phase [218]. Cardiomyocyte-specific deletion of mitochondrial transcription factor A, *Tfam*, leads to mitochondrial depolarization which increases ROS production and impairs cardiomyocyte proliferation via activation of the DNA damage response pathway leading to a severe cardiomyopathy that is embryonic lethal in mice [219]. Postnatal inactivation of *Tfam* at postnatal day 0 (P0) leads to progressive ventricular dysfunction by 6 weeks of age that can be prevented by treating with a ROS scavenger during the first postnatal week but not after the first postnatal week [219]. This suggests that there is a critical window of time in the perinatal transition during which mitochondria regulate both the cell cycle and cardiomyocyte maturation. Targeting this time frame during PSC-CM differentiation may be key to achieving in vitro maturation.

#### Quiescence versus senescence

**Fig. 5:**
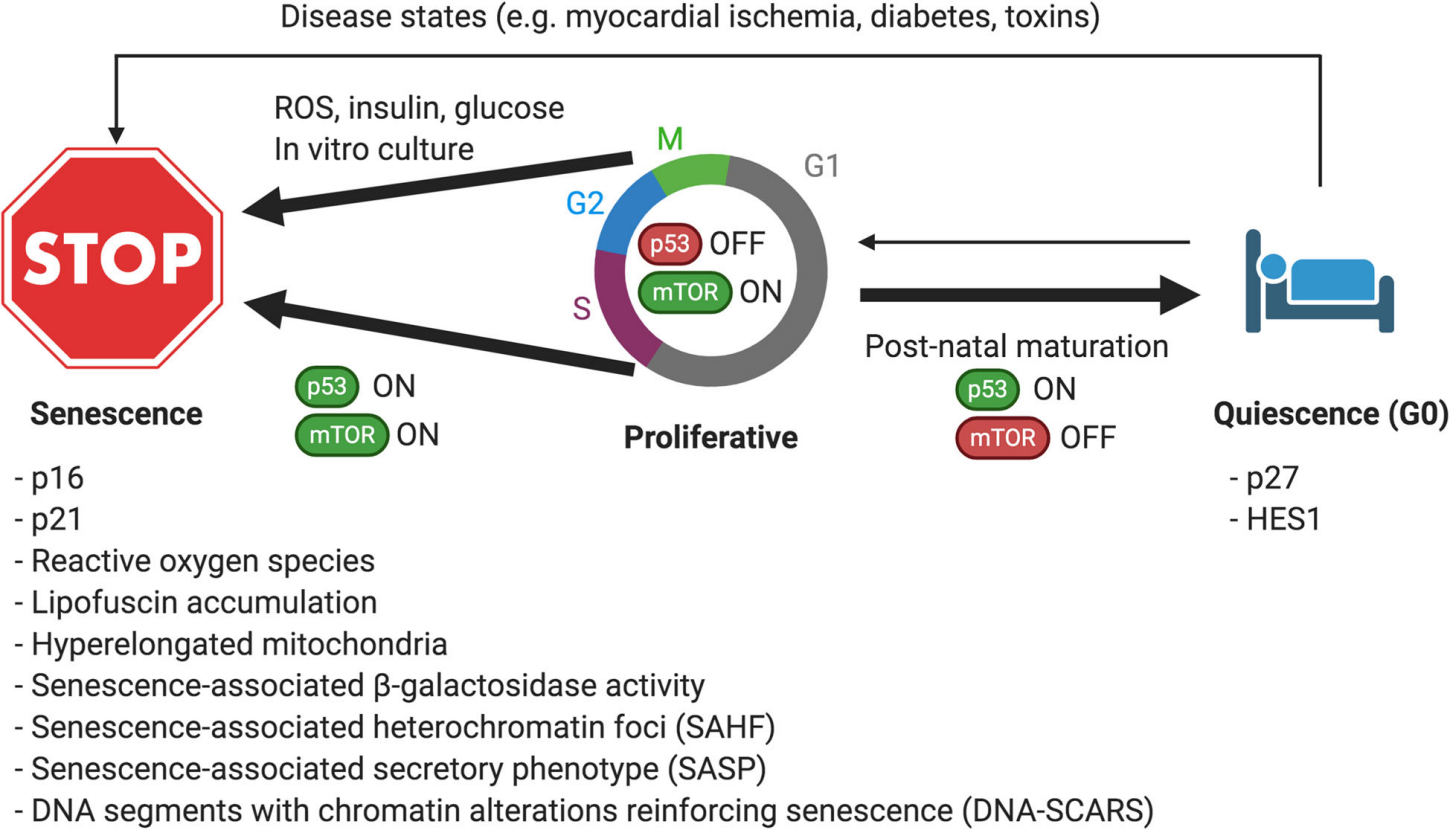
Quiescence and senescence in cardiomyocytes. Cardiomyocytes are proliferative during the fetal state but exit the cell cycle shortly after birth to enter a quiescent state. Stimuli including excessive glucose, insulin, or reactive oxygen species can induce a pathological senescent state. Cell cycle arrest with p53 upregulation leads to quiescence with inhibition of mTOR signaling but senescence with activation of mTOR signaling. In vitro culture conditions using high glucose or high insulin may also induce a senescent state and inhibition of these processes and promotion of quiescence may be important to promote cardiomyocyte maturation

Cell cycle arrest can be reversible (quiescent state) or irreversible (senescent state). Although the majority of adult cardiomyocytes do not re-enter the cell cycle, there is evidence to suggest that they are capable of proliferation under certain conditions [220], suggesting that they reside largely in a quiescent state. Features of quiescent (G_0_) cells include low RNA content, lack of proliferation markers, or increased expression of cell cycle inhibitor p27, although characteristics vary by cell type [221] (Fig. 5). In contrast, senescent cells can be arrested at the G1/S or G2/M transition [222] and exhibit elevated ROS and lipofuscin accumulation, increased SA-β-gal activity, increased expression of cell cycle inhibitors p21 or p16, senescence-associated heterochromatin foci, and have a senescence-associated secretory phenotype (SASP), secreting inflammatory factors such as interleukin-6 (IL-6), IL-8, and matrix metalloproteinases [223] (Fig. 5). Quiescence is considered a state of dormancy that is physiologic, while senescence is characteristic of aging caused by chronic damage to a cell, thus understanding what external stimuli direct cells to one or the other state may important for promote normal development.

Stimuli that promote senescence in non-myocytes include glucose [224], ROS [225], or hyperinsulinemia [226]. Therefore, standard culture conditions with supraphysiologic concentrations of glucose or insulin may promote cellular senescence in PSC-CMs. Extended culture times of PSC-CMs can improve some aspects of maturation [227], but can also increase an aging phenotype, with increased SA-β-gal activity, increased lipofuscin accumulation, and reduced mitochondrial membrane potential, which can be reversed with treatment with the antioxidant, ascorbic acid [228]. When human PSC-CMs are cultured with young, adult, or aged extracellular matrix proteins from mice, young ECM increases PSC-CM proliferation, adult ECM enhances PSC-CM function, and aged ECM produces an aging phenotype with reduced PSC-CM function and lipofuscin accumulation, increased SA-β-gal activity, and increased ROS levels [229], suggesting that extracellular factors control transitions between proliferative, quiescent, and senescent phenotypes.

We recently identified that inhibition of mTOR signaling with Torin1 can promote cardiomyocyte maturation in two-dimensional culture through induction of p53-mediated cellular quiescence and suppression of a senescent state [230]. In fibroblasts and other cell types, cell cycle arrest with p53 and mTOR activation induces senescence, while p53 upregulation with mTOR inhibition leads to quiescence [231]. Stimulators of mTOR include glucose and insulin, thus methods that demonstrate enhanced maturation of PSC-CMs with low-glucose, insulin-free media likely also inhibit mTOR activity. These pathways will need to be further tested in three-dimensional cultures as a 3D spheroid geometry can inhibit mTOR signaling alone through contact inhibition [232].

Mitochondria maintain quiescence in other cell types by suppressing glycolysis and ROS production [233]. As mentioned above, suppression of ROS may facilitate postnatal cardiomyocyte maturation, which would be supported by quiescence of postnatal cardiomyocytes. Mitochondrial morphology also changes upon transition from a proliferative to a quiescent state in other cell types [234, 235], which likely is accompanied by changes in mitochondrial function. Lysosomes also play an important role in maintaining quiescence depth and regulating mitochondrial degradation [236]. Cell culture conditions may accelerate senescence and prevent maturation; therefore, understanding what conditions promote quiescence rather than senescence may improve PSC-CM maturation.

### Epigenetics

Epigenetic regulation of gene expression via DNA methylation, histone acetylation, or microRNAs plays an important role during cardiomyocyte development [237, 238] and can influence PSC-CM differentiation and maturation [239]. DNA methylation is dynamically regulated throughout cardiomyocyte development and maturation, with increased gene expression of cardiac genes occurring with demethylation during early development and methylation occurring later to silence fetal genes [237]. Interestingly, maternal diets rich in methyl group donors (such as folate) can affect the epigenetic state of the offspring via altered DNA methylation status [240]. Population-based data have associated abnormal maternal DNA methylation with congenital heart disease in offspring, although a causal association between diet and cardiac defects has not been established [240, 241]. Epigenetic priming of differentiating PSC-CMs during the mesoderm stage promotes histone H3 lysine 9 acetylation (H3K9ac) of Notch-related genes, accelerating PSC-CM maturation [242]. Increased expression of DNA methyltransferase and increased global methylation during postnatal rat heart development suggests that decreased chromatin accessibility accompanies cardiomyocyte maturation [243]. For instance, reducing histone H3 acetylation and increasing H3 lysine 9 trimethylation (H3K9me3) downregulates expression of the fetal isoform of troponin I (ssTNI) [238]. As discussed above, microRNAs can also regulate the epigenetic state by post-transcriptional regulation of gene expression [122]. MiRNAs are involved in cardiomyocyte differentiation and maturation [244, 245]. For example, miR-1 can promote cardiomyocyte differentiation [124], while overexpression of miR-200c impairs differentiation and maturation of PSC-CMs [246]. Mitochondrial miRNAs (mitomiRs) can also regulate metabolism, energy status, and mitochondrial dynamics [247, 248].

Mitochondria play central roles in regulating the epigenetic state by producing metabolites such as acetyl-CoA and α-ketoglutarate (α-KG) that can regulate histone acetylation or DNA methylation [249, 250] and has been reviewed previously [251, 252]. Bromodomain-containing protein 4 (BRD4) is an epigenetic reader protein that recognizes acetylated chromatin sites that was recently identified to promote mitochondrial homeostasis in cardiomyocytes through GATA4-mediated upregulation of PGC-1α and PGC-1β [253, 254]. The sirtuin family of class III histone deacetylases can be activated by AMPK during low energy states to upregulate genes associated with mitochondrial biogenesis and can protect against damage from oxidative stress [255].

### Mitochondrial regulation of electrophysiological maturation

Automaticity of immature PSC-CMs is a major barrier to clinical translation due to the increased risk of ventricular arrhythmias seen when PSC-CMs are injected into large animal models of myocardial ischemia [256–258]. Imbalance of the inward rectifier K+ current (*I*_k1_) due to reduced expression of KCNJ2 and the funny current (*I*_f_) due to a relative increase in the hyperpolarization-activated cyclic nucleotide-gated (HCN) ion channels may be responsible for automaticity of PSC-CMs [259, 260]. Time-dependent changes in expression of miR-1 may be important in regulating electrophysiological maturation, as miR-1 levels are normally rise after birth, but premature overexpression of miR-1 leads to underdevelopment of the cardiac conduction system [261]. The risk of arrhythmias decreases over the first few weeks after cell delivery as PSC-CMs undergo further maturation in vivo, although the mechanisms for this remain unclear [256–258]. It is possible that changes in mitochondrial dynamics regulate this transition following exposure to the in vivo environment. Oscillations in the redox state of a cell due to dynamic nutrient metabolism can affect membrane current in cardiomyocytes [262, 263]. Neonatal rat ventricular cardiomyocytes reprogrammed into cardiac pacemaker cells with TBX18 transduction displayed automaticity, exhibited a lower metabolic demand, had smaller and more globular mitochondria, and had increased mitochondrial fission due to Opa1 downregulation compared to ventricular cardiomyocytes [264]. In addition, deletion of cyclophilin D leading to closure of the mPTP reduces the risk of ventricular arrhythmias [265]. Mutations in hydratase subunit A (HADHA) can cause sudden infant death syndrome due to cardiac arrhythmias after initiation of enteral feeds; PSC-CMs with deletion of HADHA have an immature cardiomyocyte phenotype, fragmented mitochondria with accumulation of long-chain fatty acids, and an increase in action potential duration although no change in resting membrane potential [266]. Elevated ROS can lead to mitochondrial membrane depolarization, leading to arrhythmia risk after ischemic injury [267]. Not only can mitochondria affect electrical maturation, electrical stimulation of neonatal rat ventricular cardiomyocytes can also regulate mitochondrial function via upregulation of nuclear respiratory factor 1 (NRF-1), cytochrome oxidase, and carnitine palmitoyltransferase I (CPT-I) [268].

#### Connexin 43

Connexin 43 (Cx43; encoded by GJA1) is best known as a gap junction protein that permits intercellular communication between cardiomyocytes; however, it also plays an important role during heart development [269, 270]. Global Cx43 deletion leads to right ventricular outflow tract obstruction and increased arrhythmia risk [271, 272], while cardiomyocyte-specific deletion of Cx43 leads to myocardial hypertrophy, prolongation of the QRS interval, ventricular tachyarrhythmias, and juvenile death [270, 273]. Cx43 expression is decreased in heart failure [274], while restoring Cx43 expression following myocardial infarction reduces the risk of ventricular tachycardia in animal models [275]. While the heart has high expression of the full-length, 43 kDa isoform of Cx43, it is actually the smaller, 20 kDa isoform (GJA1-20k) formed by alternative translation that is the most abundant isoform in the heart [276]. Poorly trafficked full-length Cx43 is degraded without GJA1-20k, leading to sudden cardiac death despite normal contractile function in mice [277]. GJA1-20k also associates with mitochondria to stimulate mitochondrial biogenesis via upregulation of PGC-1α and support a quiescent state with reduced ROS production [278]. In PSC-CMs, overexpression of Cx43 improves intercellular communication and improves function of the voltage-gated Na+ channels (Na_v_1.5) to increase the action potential upstroke velocity and *I*_Na_ current [279].

Nutrient availability can affect Cx43 expression, and Cx43 expression can affect both mitochondrial function and senescence. Hyperglycemic conditions downregulate expression of Cx43 while activation of AMPK increases Cx43 expression [280]. Inhibition of mTOR signaling increases expression of GJA1-20k via inhibition of cap-dependent protein translation to facilitate trafficking of the full-length isoform to the cell membrane [276]. GJA1-20k overexpression increases formation of Cx43 gap junctions, enhances mitochondrial biogenesis, and reduces ROS levels despite increased mitochondrial membrane potential and increased oxygen consumption in angiotensin II-induced cardiomyocyte hypertrophy [281]. Mitochondrial Cx43 can inhibit mPTP opening, which reduces ROS release [282]. GJA1-20k has a protective effect during oxidative stress to limit mitochondrial fragmentation in non-myocytes [283], while excessive oxidative stress and excessive mitochondrial fission can lead to degradation of Cx43 in cardiomyocytes [284].

Reduced Cx43 is considered a marker for senescence in fibroblasts [285], glomerular mesangial cells [286], and hematopoietic stem cells (HSCs) [287], although upregulation of Cx43 increases senescence of chondrocytes [288]. Cx43 prevents senescence in HSCs by forming gap junctions between HSCs and adjacent bone marrow stromal cells to transfer ROS and prevent redox damage to the HSCs [287]. Whether a similar bodyguard effect occurs between cardiomyocytes and other cell types is unclear but may also explain how ROS levels are decreased in cardiomyocytes with high Cx43 expression despite increased oxygen consumption rate.

#### Resting membrane potential

Mitochondrial dysfunction can also impair activity of the inward rectifying K^+^ ion channel, Kir2.1 (encoded by KCNJ2). Kir2.1 is largely responsible for the *I*_k1_ current which maintains the resting membrane potential of cardiomyocytes [289]. Skeletal myoblasts with depletion of mitochondrial DNA or inhibition of mitochondrial complex III with antimycin A had reduced expression of Kir2.1 leading to membrane depolarization, suggesting that impaired oxidative phosphorylation can have direct adverse effects on resting membrane potential [290]. AMPK activation downregulates Kir2.1 [291], and we recently found that sustained activation can enhance maturation of contractile and metabolic features of PSC-CMs but also reduces expression of KCNJ2 [292]. In contrast, in neurons, mTOR signaling increases expression of HCN channels [293]. Dysregulation of AMPK and mTOR signaling may affect expression levels of Kir2.1 and HCN4 in cardiomyocytes, leading to cardiomyocyte membrane depolarization.

## Summary

**Fig. 6:**
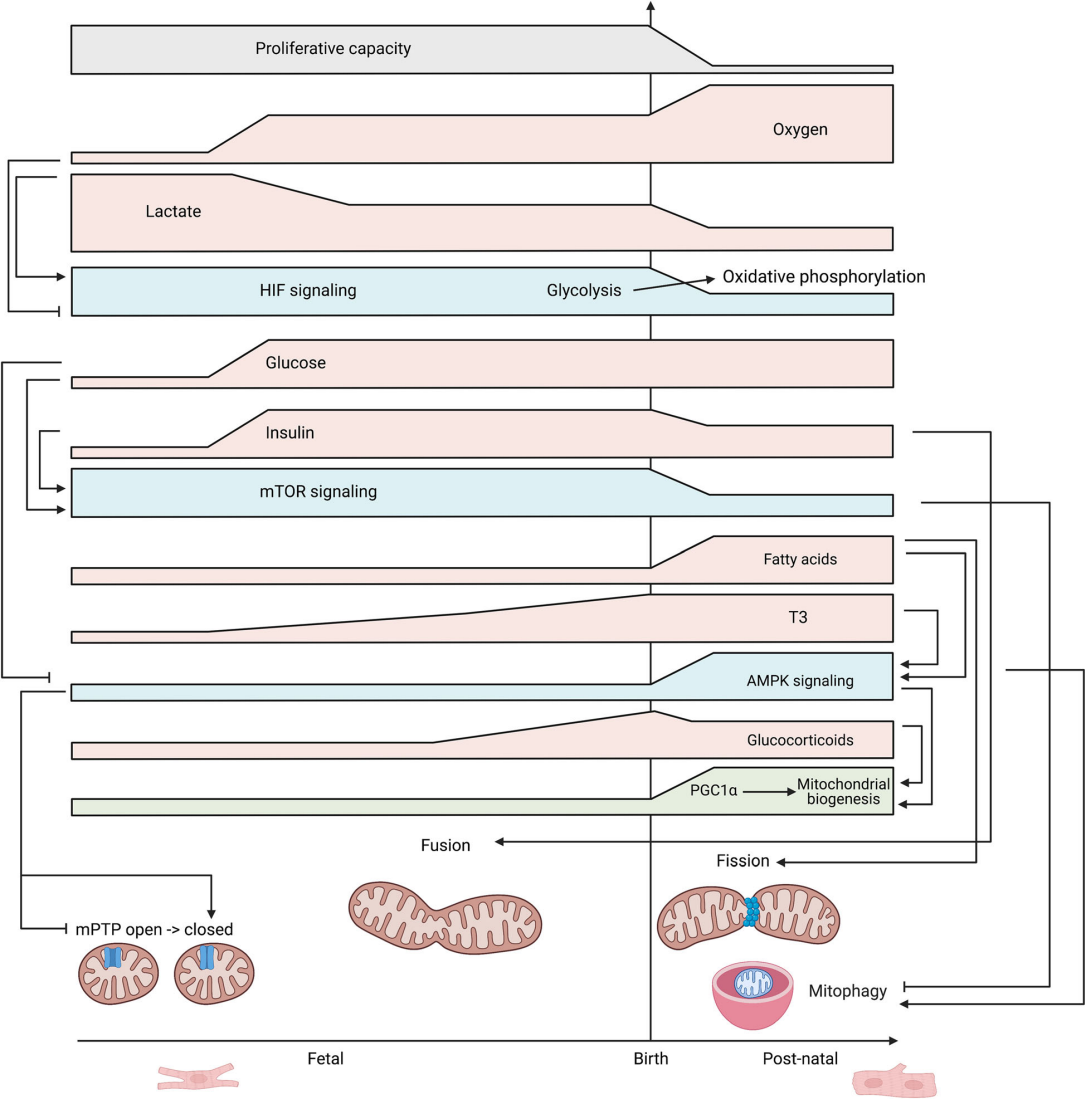
Summary of time course of metabolic changes affecting mitochondria and cardiomyocyte maturation. Dynamic changes in levels of substrates (pink) (oxygen, lactate, glucose, insulin, fatty acids, T3, glucocorticoids) affect the metabolic sensor signaling pathways (blue) (hypoxia-inducible factors (HIFs), mechanistic target of rapamycin (mTOR), AMP-activated protein kinase (AMPK)) during fetal and postnatal cardiomyocyte development. These metabolic sensors can regulate mitochondrial function and coordinate mitochondrial dynamics throughout development. PGC1α, peroxisome proliferator-activated receptor γ coactivator 1α; mPTP, mitochondrial permeability transition pore

Metabolic signals can alter mitochondrial dynamics and function, and this may precede downstream regulation of cardiomyocyte maturation. Crosstalk between key nutrient sensing systems including mTOR and AMPK can control mitochondrial behavior leading to alterations in ROS levels, cell cycle arrest, and epigenetic changes. Mimicking key developmental transitions that directly involve mitochondria such as at ~ E9.5 in mice with closure of the mPTP or at birth with transition to enteral nutrition may be critical to enhance PSC-CM maturation in vitro. Interplay between multiple metabolic factors affects mitochondrial dynamics and cardiomyocyte maturation in a time-dependent manner throughout development (Fig. 6). Mitochondria may be the conductors that drive cardiomyocyte maturation by responding to nutrient signals, dynamically altering respiratory capacity and ROS production, and initiating transcriptional and epigenetic changes guiding cardiomyocyte maturation.

## Data Availability

Not applicable.

## Abbreviations

AMP: Adenosine monophosphate
AMPK: AMP-activated protein kinase
ATP: Adenosine triphosphate
ATRA: All-trans retinoic acid
BCAA: Branched chain amino acids
CAMKII: Ca^2+^/calmodulin-dependent protein kinase II
Cx43: Connexin 43
DMEM: Dulbecco’s modified Eagle’s medium
ERR: Estrogen-related receptor
ESC: Embryonic stem cell
FDG: Fluorodeoxyglucose
HADHA: Hydratase subunit A
HIF: Hypoxia-inducible factor
IGF1: Insulin-like growth factor 1
IMM: Inner mitochondrial membrane
KLF15: Krüppel-like factor 15
LDH: Lactate dehydrogenase
LXRα: Liver X receptor α
MAO-A: Monoamine oxidase-A
MHCα: Myosin heavy chain α
miRNA: MicroRNA
MLC2v: Myosin light chain 2v
mPTP: Mitochondrial permeability transition pore
mTOR: Mechanistic target of rapamycin
mTORC: mTOR complex
NRG1: Neuregulin-1
O-GlcNAc: O-linked N-acetylglucosamine
OMM: Outer mitochondrial membrane
P70S6K: p70 ribosomal protein S6 kinase activity
PGC-1α: PPARγ coactivator 1α
PPARγ: Peroxisome proliferator-activated receptor γ
PI3K: Phosphoinositide 3-kinase
PSC: Pluripotent stem cells
PSC-CMs: Pluripotent stem cell-derived cardiomyocytes
RARE: Retinoic acid response element
ROS: Reactive oxygen species
RPMI: Roswell Park Memorial Institute 1640 medium
SA-β-gal: Senescence-associated-β galactosidase
SASP: Senescence-associated secretory phenotype
SERCA: Sarco-endoplasmic reticulum calcium-ATPase
TNNT2: Cardiac troponin T
